# Feasibility and safety of combined laparoscopic and transvaginal oocyte retrieval in a woman with vaginal recurrence of cervical adenocarcinoma: a case report

**DOI:** 10.3389/frph.2023.1295939

**Published:** 2024-01-08

**Authors:** Caroline Ingold, Paula Andrea Navarro, Renato de Oliveira, Caio Parente Barbosa, José Carlos Sadalla, Giuliano Bedoschi

**Affiliations:** ^1^ Human Reproduction and Genetics Center, Faculdade de Medicina do ABC, Santo André, SP, Brazil; ^2^ Department of Gynecology and Obstetrics, Reproductive Medicine Division, Ribeirao Preto Medical School, University of Sao Paulo, Ribeirao Preto, Brazil; ^3^ Núcleo de Cirurgia Oncológica—Mastologia e Ginecologia, Hospital Beneficência Portuguesa de São Paulo, São Paulo, Brazil

**Keywords:** fertility preservation, oocyte retrieval, cryopreservation, uterine cervical neoplasms, laparoscopy

## Abstract

**Introduction:**

Oocyte cryopreservation is an established technique for fertility preservation in women diagnosed with cancer. However, some clinical scenarios may preclude the commonly used transvaginal approach to oocyte retrieval. In such cases, a laparoscopic approach may be required. Here, we report the feasibility and safety of a combined laparoscopic and transvaginal approach for oocyte retrieval in a woman with vaginal recurrence of cervical adenocarcinoma. This approach allowed for oocyte cryopreservation prior to cancer treatment, representing a novel application in this clinical context.

**Methods:**

A 31-year-old woman with endocervical adenocarcinoma underwent laparoscopic radical hysterectomy and pelvic lymph node dissection. She presented with vaginal recurrence and was referred for fertility preservation by oocyte cryopreservation before chemotherapy and radiotherapy/brachytherapy. Ovarian stimulation was initiated with a gonadotropin antagonist protocol combined with aromatase inhibitors, and oocyte retrieval was performed with a combined laparoscopic and transvaginal approach.

**Results:**

A total of 18 oocytes were retrieved and 10 mature oocytes were cryopreserved. Peritoneal fluid cytology was negative for malignancy. The patient underwent chemotherapy and radiotherapy/brachytherapy and was disease-free after oocyte retrieval.

**Conclusion:**

The combined laparoscopic and transvaginal approach for oocyte retrieval emerges as a practical and efficacious method for fertility preservation in cases of cervical adenocarcinoma with vaginal recurrence. Further comprehensive studies are warranted to establish the reproducibility, safety, and long-term outcomes associated with this innovative approach.

## Introduction

In Latin America, the annual incidence of cancer is estimated at 1.4 million cases, affecting approximately 7.4% (146,000) women in their reproductive age (1). Worldwide, cervical cancer is the fourth most common malignancy in women, with 30% of cases occurring in individuals of childbearing age. Treatment strategies for cervical cancer depend on the stage of the disease. Early stage interventions include procedures such as trachelectomy or hysterectomy in conjunction with pelvic lymphadenectomy. Advanced stages, on the other hand, require a comprehensive approach that includes chemotherapy followed by uterovaginal brachytherapy to achieve optimal therapeutic results (2).

The prognosis for patients with cervical cancer can vary significantly depending on the stage of the disease. Notably, patients diagnosed with bulky early-stage cervical cancer, specifically FIGO 2008 stage IB2/IIA2, experience higher rates of recurrence (34%) and lower 5-year overall survival rates (70%) when compared to those with early-stage disease, such as stage IB1/IIA1, where recurrence rates are 20%, and 5-year overall survival rates are 87% (3). Recurrence of cervical cancer remains a major problem, with a higher incidence rate within the first three years after initial diagnosis. Notably, the pelvis is the most common site for recurrence. For patients who have undergone surgical treatment, several therapeutic options are available, including the administration of chemotherapy complemented by external pelvic radiotherapy, with subsequent use of brachytherapy considered in certain cases. In specific situations, the therapeutic spectrum may also be extended to pelvic exenteration (2).

Chemotherapy and pelvic radiotherapy are known to have gonadotoxic effects on the reproductive system and have been extensively studied (4). These modalities affect the oocyte/follicle primarily through direct mechanisms, such as induction of DNA double-strand breaks, involvement of the ovarian stroma, and disruption of ovarian vessels, resulting in damage to ovarian follicular reserve (5). The clinical impact of gonadotoxicity varies among individuals and is influenced by several predictive factors, including patient age, baseline ovarian reserve, and the type and dosage of drugs administered. When treating patients of reproductive age, it is imperative to consider the potential gonadotoxic effects of these therapies and discuss fertility preservation options before initiating treatment (6).

Oocyte cryopreservation has become an established procedure for fertility preservation and offers realistic prospects of pregnancy through the use of the patient's own oocytes and the use of a surrogate uterus in the case of previous hysterectomy or pelvic radiotherapy and/or brachytherapy (7). This involves controlled hyperstimulation of the ovaries through the administration of gonadotropins, followed by the retrieval of oocytes via the transvaginal route. The transvaginal approach is currently considered the primary modality for oocyte retrieval because it is relatively less invasive and provides improved ultrasound visualization of follicles (8). However, in certain cases, such as recurrent disease or previous surgery or radiation, the transvaginal approach may not be practical (9). This case report describes successful of a combined laparoscopic and transvaginal approach for oocyte retrieval in a patient with vaginal recurrence of cervical adenocarcinoma and highlights the feasibility and efficacy of this alternative approach in similar cases.

## Case report

**Figure 1:**
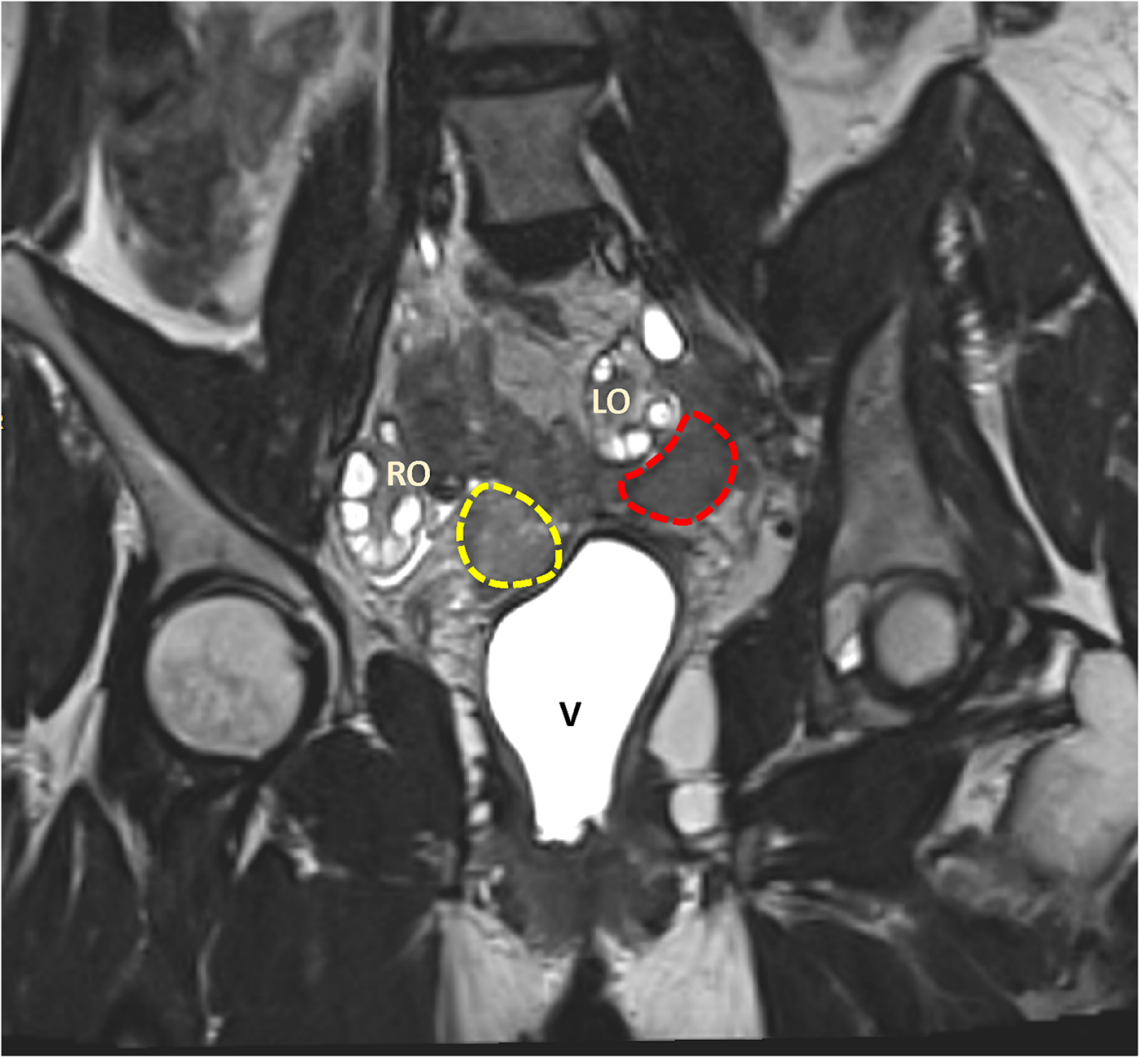
Magnetic resonance imaging (MRI) showing two infiltrative formations. The first formation is located near the right vaginal fornix (outlined in yellow), while the second is located in the left adnexal region (outlined in red). The anatomic structures shown include the right ovary (RO), the left ovary (LO), and the vagina (V).

The patient, a nulliparous woman with an unremarkable medical history, was diagnosed with stage 1B2 endocervical adenocarcinoma at the age of 30 years according to the International Federation of Gynecology and Obstetrics (FIGO 2018) staging system. Laparoscopic hysterectomy and pelvic lymphadenectomy were performed as part of the primary treatment regimen for the cancer. This was the standard procedure prior to the LACC trial (10). At the age of 31 years, the patient was diagnosed with recurrence of cervical cancer in the vaginal cuff, which was detected by magnetic resonance imaging (MRI). The MRI scan showed two infiltrative formations, one measuring 2.1 × 3.0 × 2.6 cm adjacent to the right vaginal fornix and the other measuring 2.7 × 3.0 × 2.9 cm adjacent to the lateral aspect of the left ovary (Figure 1). The latter formation involved the distal portion of the ipsilateral ureter and resulted in moderate dilatation of the upstream collecting system. The report also indicated that the lymph nodes in the abdominal and pelvic cavities were not enlarged. Systemic treatment with chemotherapy and pelvic radiotherapy was indicated. Given the recognized gonadotoxic effects of these treatments, it was decided to preserve fertility by oocyte cryopreservation before initiating concological treatment.

The patient presented to a private fertility clinic during the luteal phase of the menstrual cycle. Because the patient was suffering from surgical amenorrhea, this condition was detected by basal transvaginal ultrasound, which revealed a corpus luteum in the right ovary and an antral follicle count of 22 follicles. The ovaries were stimulated with 150 UI/day of highly purified human follicle-stimulating hormone (Fostimon®; Institute Biochimique SA, Lugano, Switzerland) and 75 UI/day human menopausal gonadotropins (Merional®, Institute Biochimique SA, Lugano, Switzerland) in combination with letrozole 5 mg/day (Letrozol, 2.5 mg, Eurofarma, Sao Paulo, Brazil), starting on the next day of the initial appointment. The gonadotropin-releasing hormone (GnRH) antagonist ganirelix acetate (Orgalutran®, 250 µg, Organon, Vetter Pharma-Fertigung GmbH & Co. KG, Ravensburg, Germany) was administered daily from the sixth day of stimulation. Oocyte maturation was induced on day 11 of ovarian stimulation with 0.3 mg of the GnRH agonist triptorelin (Gonapeptyl®, 0.1 mg, Ferring GmbH, Kiel, Germany). At the time of ovulation induction, the patient had seven follicles with a mean diameter of ≥18 mm and an estradiol peak of 583 pg/ml.

Because of the recurrence of cancer in the vaginal vault, transvaginal access to the right ovary was considered technically difficult, raising concerns about the possibility of cancer dissemination and hemorrhagic complications. Therefore, the decision was made to perform a laparoscopic oocyte retrieval from the right ovary and a transvaginal ultrasound-guided oocyte retrieval from the left ovary, exactly 35 h after triggering. Abdominal access was obtained with a 10-mm trocar through the umbilicus, whereas a 5-mm trocar was placed in the left lower quadrant to allow optimal mobilization of the ovary. For access and retrieving follicles in the right ovary, the Veress needle was inserted into the right lower quadrant, creating a channel for passage of the oocyte retrieval needle. Initial inspection revealed no intra-abdominal adhesions, and an upper abdominal examination confirmed normality.

A total of 18 oocytes were collected and denuded, with 8 oocytes obtained from the right ovary and 10 oocytes obtained from the left ovary. Ten mature oocytes were vitrified within 2 h of oocyte retrieval using the Cryotop® device (Kitazato BioPharma, Fuji, Japan) in combination with the Cryotech® vitrification method (Repro-Support Medical Research Centre, Tokyo, Japan) according to the manufacturer's protocol.

**Figure 2:**
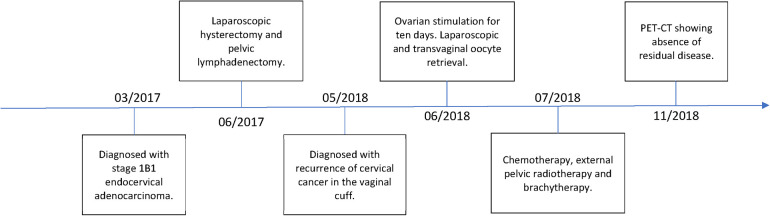
Historical and current information from this episode of care organized as a timeline.

During the laparoscopic procedure, cytological analysis of the peritoneal fluid was performed, which revealed no evidence of malignant cells. The patient did not require any intraoperative hemostasis procedures, such as coagulation or suturing on the right ovary. Likewise, the patient did not experience any procedure-related complications. After laparoscopic oocyte retrieval, the patient underwent placement of a double-J stent to relieve the urinary tract obstruction. She then received six cycles of platinum-based chemotherapy and 25 fractions of external pelvic radiotherapy, followed by three fractions of high-dose brachytherapy. The patient responded well to treatment and achieved complete remission of her cervical cancer (Figure 2).

## Discussion

Cervical cancer has a significant impact on women of reproductive age, underscoring the need to carefully consider the impact of various treatment modalities on fertility. Therapies that target the pelvic region, particularly radiotherapy, may affect ovarian function because the ovaries are inevitably exposed to radiation in this region. To mitigate this risk, ovarian transposition or oophoropexy is a viable option. In this surgical procedure, the ovaries are relocated to a non-irradiated site to reduce the risk of radiation-induced ovarian insufficiency and preserve ovarian function (11). However, it is important to acknowledge that despite this intervention, the guarantee of future fertility or successful pregnancies is not absolute. In particular, chemotherapy, especially the use of cisplatin, carries a significant risk of gonadotoxicity, which leads to impaired ovarian function in approximately 30% of patients (4). Therefore, women of reproductive age undergoing chemotherapy, radiotherapy, and/or brachytherapy are strongly advised to consider oocyte cryopreservation before starting treatment to protect their fertility (6).

Timely initiation of ovarian induction treatment is important to avoid delay of chemotherapy. Therefore, strategies such as starting the stimulation cycle in the luteal phase have been proposed to achieve this goal (12), with recent study demonstrating comparable number of mature oocytes and embryos cryopreserved when compared to standard-start-controlled ovarian stimulation (13). In addition, the use of a GnRH agonist to induce ovulation has been shown to reduce the risk of ovarian hyperstimulation syndrome (14, 15). Co-administration of letrozole has been shown to significantly lower serum estradiol levels compared with conventional ovarian stimulation protocols, making treatment safer (16). Such an approach is particularly important in patients with hormone receptor-positive tumors, in whom high estrogen levels can promote tumor growth and spread. This case report demonstrates the successful use of these techniques in the treatment of this patient.

The patient in this case report presented a particular challenge because the location of the tumor recurrence limited access to the right ovary and made oocyte retrieval via the conventional transvaginal route difficult. The literature indicates that in approximately 30%–50% of patients with cervical cancer, treatment fails, with local recurrence being the primary cause (17). In this case, the recurrence was located in the vaginal cuff and transvaginal access for oocyte retrieval was not considered feasible. The potential risks associated with manipulating malignant tissue near the pelvis, such as increased risk of peritoneal spread and bleeding, as well as the difficulty of accessing the right ovary due to the presence of adjacent tissue, led to the decision to use a laparoscopic approach instead.

In the past, laparoscopy was the primary method of oocyte retrieval, but over time the transvaginal approach has become the preferred method because of its lower risk of complications and minimal invasiveness. However, in cases where the transvaginal route is not feasible, laparoscopy is still a viable alternative. It should be noted that laparoscopic oocyte retrieval requires general anesthesia and may result in a longer recovery time and discomfort due to pneumoperitoneum (18).

The use of transvaginal ultrasound for oocyte retrieval has become the preferred method because it provides greater accuracy in visualizing the follicles and is noninvasive. In addition, the transvaginal method is less affected by factors such as patient obesity or difficulty in locating the ovaries. However, in certain cases, such as the one described in this case report, alternative routes of oocyte retrieval should be considered. Nevertheless, previous studies have shown that laparoscopic oocyte retrieval results in a lower yield of mature oocytes and a lower fertilization rate (18).

In agreement with the study by Doyle et al. (19), the successful retrieval of ten mature oocytes in this case gives the patient a favorable 60% probability of having at least one viable child. Given the patient's history of total hysterectomy, the use of these oocytes depends on surrogacy or uterine transplantation as viable options. Advances in assisted reproductive technologies and evolving surgical techniques offer optimistic prospects for the patient to attain motherhood through these alternative routes and underscore the possibility of fulfilling her desire for biological motherhood.

In Brazil, surrogacy is regulated by specific guidelines established by the Brazilian Federal Medical Council. According to Resolution 2.320/2022, surrogacy may only be performed by a relative of one of the partners in a consanguineous relationship up to the fourth degree who has at least one living child. In addition, surrogacy must not be performed for profit or commercial purposes, and the fertility clinic must not facilitate the selection of the recipient. If the recipient is married or in a committed relationship, the written consent of the spouse or partner is required. In cases where it is not possible to meet these requirements, permission may be obtained from the Federal Medical Council. These regulations are intended to ensure the ethical and safe use of surrogacy in Brazil (20).

## Conclusion

In conclusion, cervical cancer is a significant health problem for women of childbearing age and it is important to consider the potential gonadotoxicity associated with treatments such as pelvic irradiation and chemotherapy. In this case report, the patient faced challenges due to the anatomic location of the tumor recurrence, which limited access to the right ovary and posed difficulties in performing oocyte retrieval via the transvaginal route. Therefore, it was decided to use an alternative technique for oocyte retrieval, namely the laparoscopic method. However, it should be noted that laparoscopic oocyte retrieval may yield a lower number of mature oocytes and a lower fertilization rate than transvaginal retrieval. Despite the challenges in this case, the patient still had a 60% chance of having a live child after retrieval of ten mature oocytes. In addition, the patient had the possibility of pregnancy through surrogacy. It is also important to note the regulations that apply to surrogacy in Brazil.

The combined laparoscopic and transvaginal approach to oocyte retrieval appears to be safe and effective in women with vaginal recurrence of cervical adenocarcinoma who need to preserve their fertility before cancer treatment. Larger studies are needed to confirm the feasibility and safety of this approach.

## Data Availability

The original contributions presented in the study are included in the article/Supplementary Material, further inquiries can be directed to the corresponding author.
